# Acute respiratory infection and its associated factors among children under-five years attending pediatrics ward at University of Gondar Comprehensive Specialized Hospital, Northwest Ethiopia: institution-based cross-sectional study

**DOI:** 10.1186/s12887-020-1997-2

**Published:** 2020-02-28

**Authors:** Henok Dagne, Zewudu Andualem, Baye Dagnew, Asefa Adimasu Taddese

**Affiliations:** 1 Department of Environmental and Occupational Health and Safety, Institute of Public Health, University of Gondar, P.O.Box 196, Gondar, Ethiopia; 2 Department of Human Physiology, School of Medicine, University of Gondar, P.O.Box 196, Gondar, Ethiopia; 3 Department of Epidemiology and Biostatistics, Institute of Public Health, University of Gondar, P.O.Box 196, Gondar, Ethiopia

**Keywords:** Acute respiratory infection, Under-five years, Ethiopia

## Abstract

**Background:**

Acute respiratory infection is manifested by cough accompanied by short rapid breathing which may be associated with death especially when there are other co-morbidities. From an estimated 5.4 million children under –five years that died in 2017—roughly half of those deaths occurred in sub-Saharan Africa and acute respiratory infection contributed to the highest number of deaths. The current study aimed at evaluating the prevalence of, and risk factors associated with, acute respiratory infection hospitalization in under-five years children hospitalized at the University of Gondar Comprehensive Specialized Hospital.

**Method:**

An institution-based cross-sectional study was carried out from May 01/2019 to July 10/2019. After the selection of participants using simple random sampling, face to face interview was performed using a semi-structured pre-tested questionnaire. Data were also extracted from medical registration charts. We used EPI Info 7 for data entry and exported into SPSS 21 for analysis. Results were presented by simple frequency, percentage and mean for descriptive variables. Binary logistic regression analysis was used to test the association of covariates and outcome variable. Variables with a *p* < 0.2 during the bivariable binary logistic regression analysis were included in the multivariable logistic regression analysis. Variables with *p* < 0.05 were considered as significantly associated with acute respiratory infection. This study is reported following the Strengthening the Reporting of Observational Studies in Epidemiology guideline.

**Results:**

Four hundred and twenty-two under-five years’ children attending the Pediatrics ward were included in this study. The prevalence of acute respiratory infection among under-five years’ children in this study was 27.3%. Children aged below 12 months (AOR:3.39, 95% CI: 1.19, 9.65), maternal age of 16 to 27 years (AOR: 1.95, 95% CI: 1.03, 3.70), maternal age of 28 to 33 years (AOR: 2.73, 95% CI: 1.40, 5.34), lack of maternal awareness of handwashing (AOR: 2.79, 95% CI: 1.15, 6.76), rural residence (AOR:2.27, 95% CI: 1.18, 4.39), and lack of meningitis (AOR: 0.22, 95% CI: 0.08, 0.55), were significantly associated with acute respiratory infection.

**Conclusion:**

Acute respiratory infection was common among children under-five years. Child and maternal age, residence and maternal hand hygiene information were significant factors identified to be associated with an acute respiratory infection.

## Background

Acute respiratory infection (ARI) is manifested by cough accompanied by short rapid breathing which may be associated with death especially when there are other co-morbidities [1], even though a significant decline has been achieved over the past two decades [2]. From an estimated 5.4 million under-five children that died in 2017—roughly half of those deaths occurred in sub-Saharan Africa and ARIs contributed to the highest number of deaths [3]. ARIs are among the leading causes of morbidity and mortality among children under-five years worldwide [3]. Mortality due to ARI is significantly varied across regions [4]. In 2010, global burden disease reported that more than 12 million children with severe ARI were admitted to hospitals every year worldwide [5]. ARI accounts for up to 50% of visits of children to health facilities globally [6].

Pneumonia accounts for the death of approximately 2400 under-five years children a day [7]. ARIs are responsible for approximately 70% of under-five years of childhood morbidities in developing countries [8]. A study conducted to assess the prevalence of acute lower respiratory infections (ALRIs) among children under-five years from 28 sub-Saharan African countries revealed the overall prevalence of ARI for all the countries was 25.3% [9]. In Ethiopia, 7 % of under-five years had symptoms of ARI in the 2 weeks before the Ethiopian Demographic and Health survey and three out of 10 of these children sought treatment [10]. The under-five years’ mortality rate in Ethiopia is 67 deaths per 1000 live births [10] .

Several factors predispose children under five years of age for ARIs. These factors may be attributed to child factors such as age [11–15] and female sex [16], maternal factors such as lower age [11, 13], unemployment [11, 13] and lower educational status [16, 17] environmental-related factors such as urban residence [17], rural residence [18], wet season [19–21] and co-morbid diseases [14, 22, 23]. There is a paucity of studies regarding the prevalence and associated factors of ARI among hospitalized under-five years children in Ethiopia even though few community-based cross-sectional studies [24–26] have been undertaken in to assess the prevalence and associated factors of ARIs among under-five years children. The current study, therefore, is aimed at assessing the prevalence and associated factors of ARI among under-five years’ children hospitalized in the Pediatric ward at University of Gondar Comprehensive Specialized Hospital, northwest Ethiopia.

## Methods

### Study design and settings

The current study was conducted at the University of Gondar Comprehensive Specialized Hospital among randomly selected under-five years children who were admitted at the Pediatrics ward of the Hospital from May 01/2019 to July 10/2019. The mothers of the children were respondents in this study. The Hospital is located in Gondar city, northwest Ethiopia. It is located 738 km from Addis Ababa and is serving more than 5 million people annually. In 2018, the Hospital had 1040 health care professionals, 580 beds in five different inpatient departments and 14 wards, and 14 different units giving outpatient services to customers [27, 28].

### Sample size determination and sampling technique

The sample size was calculated using a single population proportion formula [29] assuming; prevalence of ARI (p) = 50% to allow maximum variation (as there was no previous institutional-based study in the country about the proportion of under-five years hospitalized children with ARIs), 95% confidence level, z = the standard normal tabulated value, and α = level of significance and margin of error (d) =0.05

$$ n=\frac{{\left({Z}_{\raisebox{1ex}{$\alpha $}\!\left/ \!\raisebox{-1ex}{$2$}\right.}\right)}^2p\left(1-p\right)}{d^2}=\frac{(1.96)^20.5\left(1-0.5\right)}{0.05^2}=384 $$

After adding expected oversampling of 10% for unexpected events, the final total sample size was 422. Study participants were selected using a computer-generated simple random sampling technique using their medical registration number. Sampling was taken on daily basis and children were selected from a random number list for each day. Whenever parents did not consent the next number was taken.

### Eligibility criteria

Under-five years who visited the Paediatrics ward at University of Gondar Comprehensive Specialized Hospital at time of data collection period in which their mothers consented to participate were included whereas children whose mothers or caretakers refused to take part in the study due to different reasons were excluded.

### Data collection instrument and quality control

A pre-tested, semi-structured questionnaire containing socio-demographic variables on maternal and child factors and extraction tool to review chart was used. Interviews and chart reviews were undertaken by three BSc Nurse Professionals. Training about the data collection tool, techniques, the purpose of the study, data extraction tool and ethical issues was given for data collectors. The questionnaire was validated for content and reliability analysis was performed based on the pretest result on 20 individuals. We have also made corrections for ambiguities before actual data collection. Comments were obtained from each participant and based on their recommendations, the questionnaire was updated. The internal consistency was analyzed using Cronbach’s α coefficient [30]. The Cronbach’s α results were 0.7 for knowledge, 0.73 for attitude, 0.87 for practice and 0.78 overall results. According to George, this is acceptable internal consistency [31].

### Study variables

The dependent variable in the current study was children’s acute respiratory infection. The independent variables were child-related (age, sex, residence, comorbidities (diarrhoea, meningitis and malnutrition), maternal factors (age, education, information, knowledge, attitude and practice of handwashing)).

### Measurement of variables

The variable of interest in this study was ARI occurring in under-five years children. Presence or absence of ARI was determined by the health professionals with any one or combination of symptoms and signs like cough, sore throat, rapid breathing, noisy breathing, chest indrawing, at any time in the last 2 weeks and the status is taken directly from the chart review. Meningitis assessment was based on physicians assessment. Diarrhoea was defined as having three or more loose or watery stools within 24 h [32]. Malnutrition was determined by anthropometric measurement of mid-upper arm circumference, weight for age and height for age [33]. The maternal knowledge, attitude and practice regarding handwashing were determined by asking the mothers knowledge, attitude and practice questions. Since the data for all of the three variables were normally distributed, we used mean to dichotomize maternal handwashing knowledge, attitude and practice as good or poor. Study subjects that scored at the mean or above the mean on maternal knowledge, attitude and practice questions were considered having good knowledge, attitude and practice. The maternal age was categorized based on the quartile range.

### Data processing and analysis

The data were entered using Epi-Info version 7 and analyzed using SPSS statistical package version 21.0. All assumptions for binary logistic regression were checked.

To determine predictor variables for ARI, a binary logistic regression model was fitted and variables at a *p-*value < 0.2 during the bi-variable analysis were included in the multivariable analysis.

Finally, variables found to be significant at a *p-*value < 0.05 in the final model were declared as predictors. Crude odds ratios (COR) and adjusted odds ratios (AOR) with 95% confidence interval were reported. Hosmer and Lemeshow goodness- of -fit test (*p* > 0.05) was used to check model fitness. The report was prepared based on the Strengthening the Reporting of Observational Studies guideline.

## Results

### Sociodemographic characteristics of study participants

Four hundred and twenty-two under-five years’ children were included in the current study.

Most (238/422, 56.4%) of the children resided in an urban setup and 221 (50.0%) were male. The prevalence of ARI, diarrhea and malnutrition were 27.3, 30.1 and 24.4%, respectively (Table 1).

**Table 1:** Characteristics of under-five years children admitted in University of Gondar Comprehensive Specialized Hospital Pediatrics ward, northwest Ethiopia, 2019 (*n* = 422)

Variables Categories	Frequency	Percent
Residence		
Rural	184	43.6
Urban	238	56.4
Child sex		
Female	211	50
Male	211	50
Child age		
Below 12 months	31	7.3
12–23 months	55	13
24–35 months	96	22.7
36–47 months	145	34.4
48–59 months	95	22.5
Diarrhoea		
Yes	127	30.1
No	295	69.9
Malnutrition		
Yes	103	24.4
No	319	75.6
Acute respiratory infection		
Yes	115	27.3
No	307	72.7

### Factors associated with ARI

The bivariable analysis revealed that child and maternal age, residence, maternal educational status and information about handwashing, knowledge and practice about handwashing were variables with a *p*-value < 0.2, and these variables were included in the multivariable logistic regression model. Child and maternal age, residence, and maternal information about handwashing were significantly associated with ARI among under-five years in the final model.

Children below 12 months had a 3.39-fold (AOR: 3.39, 95% CI: 1.19, 9.65) increased adjusted odds of ARI hospitalization. Under-five years children of mothers aged 16 to 27 and 28 to 33 years had a 1.95 (AOR: 1.95, 95% CI: 1.03, 3.70) and a 2.73(AOR: 2.73, 95% CI: 1.40, 5.34) adjusted odds of ARI hospitalization compared to those whose mothers older than 42 years. The adjusted odds of ARI was 2.27-fold (AOR: 2.27, 95% CI: 1.18, 4.39) higher in children residing in a rural setting compared to those from urban setup. Similarly, children with meningitis had 78% reduced adjusted odds of ARI hospitalization (AOR: 0.22, 95% CI: 0.08, 0.55) compared to those without meningitis. Children whose mothers reported lack of knowledge about handwashing had a 2.79-fold (AOR: 2.79, 95% CI: 1.15, 6.76) increased odds of ARI hospitalization compared to those whose mothers knew about handwashing **(**Table 2**).**

**Table 2:** Factors associated with acute respiratory infection among Under-five years children attending Pediatrics ward at University of Gondar Comprehensive Specialized Hospital northwest Ethiopia, 2019 (n = 422)

Variables	Category	ARI		COR(95% CI)	p-value	AOR (95% CI)
		Yes (%)	No (%)			
Child sex	Male	58(50.4)	153(49.8)	1		
	Female	57(49.6)	154(50.2)	0.98(0.64, 1.50)	0.913	–
Maternal age (years)	16–27	27(23.5)	79(25.7)	1.79(0.90,3.58)	0.097	1.95(1.03,3.70)*
	28–33	36(31.3)	73(25.8)	2.59(1.33,5.04)	0.005	2.73(1.40,5.34)**
	34–42	36(31.3)	71(23.1)	2.66(1.36,5.19)	0.004	1.12(0.52,2.42)
	> 42	16(13.9)	84(27.4)	1	1	1
Current maternal marital status	not married	45(39.1)	134(43.6)	1	1	
	Married	70(60.9)	173(56.4)	1.20(0.78, 1.87)	0.403	–
Residence	Urban	89(77.4)	149(48.5)	1	1	1
	Rural	26(22.6)	158(51.5)	3.63(2.22,5.93)	0.000	2.27(1.18,4.39)*
Maternal education	Illiterate	17(14.8)	105(34.2)	0.33(0.19,0.59)	0.000	0.63(0.30,1.30)
	Literate	98(85.2)	202(65.8)	1	1	
Meningitis	No	109(28.9)	268(71.1)	1		
	Yes	6(13.3)	39(86.7)	0.38(0.16,0.92)	0.032	0.22(0.08,0.55)**
Mothers have information about hand washing	No	7(9.3)	68(90.7)	4.41(1.96, 9.92)	0.000	2.79(1.15,6.76)*
	Yes	108(31.2)	238(68.8)	1		
Child age (months)	Below 12	12(10.4)	19(6.2)	2.37(0.99, 5.68)	0.053	3.39(1.19,9.65)*
	12–23	19(16.5)	36(11.7)	1.98(0.94,4.16)	0.072	2.17(0.95,5.00)
	24–35	28(24.3)	68(22.1)	1.54(0.80,2.99)	0.198	1.82(0.86,3.82)
	36–47	36(31.3)	109(35.5)	1.24(0.67,2.30)	0.499	1.37(0.69,2.72)
	48–59	20(17.4)	75(24.4)	1	1	1
Maternal handwashing practice	Good	86(32.8)	176(67.2)	1		1
	Poor	29(18.1)	131(81.9)	0.45(0.28,0.73)	0.001	0.57(0.31,1.05)
Maternal handwashing knowledge	Good	89(31.8)	191(68.2)	1		1
	Poor	26(18.3)	116(81.7)	0.48(0.29,0.79)	0.004	0.94(0.50,1.78)
Maternal handwashing attitude	Good	65(56.5)	166(54.1)	1	1	
	Poor	50(43.5)	141(45.9)	0.91(0.59, 1.39)	0.653	–

**Significant at *p*-value< 0.01, *Significant at *p*-value< 0.05, Hosmer and Lemshow =0.517 *COR* crude odds ratio, *AOR* adjusted odds Ratio, *CI* confidence interval

## Discussion

The current study aimed at evaluating the prevalence of, and risk factors associated with, ARI hospitalization in under-five years children hospitalized at the University of Gondar Comprehensive Specialized Hospital. The proportion of under-five years children with ARI in this study was 27.3 95% CI (23.2–31.5%). This is in line with the overall prevalence of ARIs among children under-five years from 28 Sub-Saharan African countries (25.3%) [9], previous studies in Gondar (26.3%) [24] and Addis Ababa (23.9%) [26]. However, it is lower than prevalence of ARI reported from India (41.6%) [34], Cameroon (54.7%) [35], Nigeria (64.9%) [36], Kenya (69.7%) [37], and Bangladesh (70%) [38]. The current prevalence is slightly higher than a study conducted in southern Ethiopia (21%) [25]. This variations in the proportions of ARI could be as a result of differences in study populations, study settings (community-based versus institutional-based), age categories studied, the method used to assess the outcome variable, comorbidities and variations in the study period and season of the study.

From the factors tested in the current study; child age, maternal age, residence, and maternal handwashing information were significantly associated with ARIs among under-five years children attending the Pediatric ward of the University of Gondar Comprehensive Specialized Hospital.

Child age was associated with ARI hospitalization. The odds of developing ARI was higher among children below 12 months of age as compared to those aged above 48 months. This was in line with a previous study [39]. Higher risk of ARI among lower age children might be due to less developed immunity [40]. However, it was inconsistent with another study [35].

Maternal age was associated with children’s ARI status. The adjusted odds of developing ARI was 1.95 and 2.73-folds higher among children having mothers aged 16 to 27 and 28–33 years, respectively. This was in line with previous studies [11, 41–43]. Maternal age was not associated in several earlier studies [9, 14, 24, 35]. The association of maternal age with ARI can be explained by mothers’ experience to give necessary and sufficient care for their children as younger mothers may be less experienced in child care services.

Children from rural setup were more prone to develop ARI in the current study which is in line with several earlier studies [12, 39, 44–46]. The probable justification for the greater ARI symptoms proportion for rural children may be due to lack of access to medical care, low socio-economic standards in rural regions [47] and most risk factors for ARI prevail in rural setup [48]. However, in other studies [23, 49], the residence was not significantly associated and Kumar et’ al reported urban residence to be a risk factor for ARI [17].

Children with meningitis infection were less likely to develop ARI. Pneumococcal meningitis incidence was found to be highly associated with the incidence of acute viral respiratory infection in previous studies [50–55]. The lesser odds of ARI among children with meningitis may be attributed to the fact that children with meningitis will receive antibiotics.

Unlike several previous studies [56–58] in the current study maternal self-defined handwashing practice and attitude were not significantly associated with ARI in the final model. This might be due to the fact that self-reported handwashing practice is from a simple cleansing to the appropriate level of recommended duration and method of handwashing. However, the lack of association in the current study is in line with a population-based study in Sweden [59]. In the current study children of mothers who have reported a lack of information about handwashing were more likely to be at higher risk of ARI.

### Limitations of the study

This study was not without limitation. Treatment and outcomes were not collected; recruitment only took place over a 3 month period, so prevalence may be different during different months of the year. The seasonality of ARI was not studied in Ethiopia previously and we had collected information within 3 months’ time and unable to see the temporal variability in the prevalence in the current study. The housing-related confounders and comorbidities such as HIV were not assessed. Hand washing practice was assessed by self-report and may be prone to social desirability bias. Besides, the cause-effect relationship cannot be established as this is a cross-sectional study. Because of the lack of sufficient institutional-based prevalence studies regarding ARI among under-five years children comparing with previous similar studies was difficult.

## Conclusion

A significant proportion of under-five years children admitted at pediatrics ward at the University of Gondar Comprehensive Specialized Hospital had ARI. Maternal and child age, residence, and maternal hand hygiene information were significant factors identified to be associated with ARI.

## Data Availability

The dataset is accessible at the corresponding author upon a reasonable request.

## Abbreviations

AOR: Adjusted odds ratio
CI: Confidence interval
COR: Crude odds ratio
EPI Info: Epidemiological information
SPSS: Statistical package for social sciences
